# *Bmi1*^+^ cardiac progenitor cells contribute to myocardial repair following acute injury

**DOI:** 10.1186/s13287-016-0355-7

**Published:** 2016-07-30

**Authors:** Iñigo Valiente-Alandi, Carmen Albo-Castellanos, Diego Herrero, Iria Sanchez, Antonio Bernad

**Affiliations:** 1 Cardiovascular Development and Repair Department, Spanish National Cardiovascular Research Center (CNIC), Madrid, Spain; 2 Immunology and Oncology Department, Spanish National Center for Biotechnology (CNB-CSIC), Madrid, Spain; 3 Unidad de Medicina Comparada, Cardiovascular Development and Repair Department, Spanish National Cardiovascular Research Center (CNIC), Madrid, Spain; 4 Current address: The Heart Institute, Cincinnati Children’s Hospital Medical Center, Cincinnati, OH USA; 5 Current address: Vivebiotech, San Sebastian, Spain

**Keywords:** Myocardial infarction, Stem cells, Bmi1, Cardiac progenitor cells

## Abstract

**Background:**

The inability of the adult mammalian heart to replace cells lost after severe cardiac injury compromises organ function. Although the heart is one of the least regenerative organs in the body, evidence accumulated in recent decades indicates a certain degree of renewal after injury. We have evaluated the role of cardiac *Bmi1*^*+*^ progenitor cells (*Bmi1*-CPC) following acute myocardial infarction (AMI).

**Methods:**

*Bmi1*^Cre/+^;*Rosa26*^YFP/+^ (*Bmi1*-YFP) mice were used for lineage tracing strategy. After tamoxifen (TM) induction, yellow fluorescent protein (YFP) is expressed under the control of *Rosa26* regulatory sequences in *Bmi1*^*+*^ cells. YFP^+^ cells were tracked following myocardial infarction. Additionally, whole transcriptome analysis of isolated YFP^+^ cells was performed in unchallenged hearts and after myocardial infarction.

**Results:**

Deep-sequencing analysis of *Bmi1*-CPC from unchallenged hearts suggests that this population expresses high levels of pluripotency markers. Conversely, transcriptome evaluation of *Bmi1*-CPC following AMI shows a rich representation of genes related to cell proliferation, movement, and cell cycle. Lineage-tracing studies after cardiac infarction show that the progeny of *Bmi1*-expressing cells contribute to de novo cardiomyocytes (CM) (13.8 ± 5 % new YFP^+^ CM compared to 4.7 ± 0.9 % in age-paired non-infarcted hearts). However, apical resection of TM-induced day 1 *Bmi1*-YFP pups indicated a very minor contribution of *Bmi1*-derived cells to de novo CM.

**Conclusions:**

Cardiac *Bmi1* progenitor cells respond to cardiac injury, contributing to the generation of de novo CM in the adult mouse heart.

**Electronic supplementary material:**

The online version of this article (doi:10.1186/s13287-016-0355-7) contains supplementary material, which is available to authorized users.

## Background

Myocardial infarction leads to an irreversible loss of cardiac myocytes, which compromises cardiac function. Although adult mammals are believed to have a very limited capacity to replace cardiomyocytes (CM), the traditional view of a terminally differentiated organ has recently been challenged by evidence of low intrinsic CM turnover in the adult mouse [1] and human hearts [2]. Estimated rates of CM turnover nonetheless vary greatly [3], and the underlying mechanisms [1, 4] and the origin of new cells contributing to adult cardiomyocyte turnover after injury remain unclear [5]. The main concepts to explain heart turnover are based on proliferation of CM [6–8] or the potential of cardiac progenitor cells (CPC) that are defined primarily by surface markers such as c-KIT [9] or SCA-1 (also known as Ly6a) [10, 11]. The diversity of findings for these populations and the absence of specific, definite markers to identify CPC have given rise to notable controversy.

*Bmi1*, a member of the Polycomb repressive complex 1 (PRC1), has a central role during self-renewal and maintenance of several adult stem cell compartments [12–14]. High *Bmi1* expression defines a population of adult resident cardiac progenitor cells (*Bmi1*-CPC) that are able to generate de novo CM during homeostasis in the adult heart [15]. Here we show that *Bmi1*-CPC have a stemness genetic profile, are activated, and contribute to de novo CM after acute myocardial infarction (AMI).

## Methods

### Bmi1IRESCreER mice and tamoxifen administration

Males and female double heterozygous *Bmi1*^CreER/+^;*Rosa26*^YFP/+^ (*Bmi1*-YFP) mice, generated by crossing the *Bmi1*^CreER/+^ strain with *Rosa26*^YFP/+^ reporter mice, received intraperitoneal (i.p.) tamoxifen (TM) injections (Sigma) between postnatal days 30 (P30) and P60. For neonatal studies, pregnant *Bmi1*^CreER/CreER^ x *Rosa26*^YFP/YFP^ mothers were TM-induced at 17.5 and 18.5 days postcoitum. TM was dissolved in corn oil (Sigma; final concentration 20 mg/ml), and mice received TM every 24 h on three consecutive days (9 mg/40 g body weight; each day). The ethics committees of the Spanish Cardiovascular Research Center (CNIC) and the Spanish National Center for Biotechnology (CNB) approved the animal studies.

### Immunodetection analysis

Heart immunohistochemistry was performed as described [6]. Specific yellow fluorescent protein (YFP) detection with anti-green fluorescent protein (GFP) antibody was confirmed by immunofluorescence analysis of two control conditions (heart sections from TM-injected *Rosa26*^YFP/+^ and non-injected *Bmi1*-YFP mice (*Bmi1*-YFP^NI^)); no signal was observed in either [15]. For immunodetection, sections were fixed in 2 % paraformaldehyde (PFA) and rinsed in PHEM buffer (25 mM Hepes, 10 mM EGTA, 60 mM PIPES, 2 mM MgCl_2_; all from Sigma). Slides were rinsed in blocking buffer (0.5 % porcine skin gelatin, 0.1 % bovine serum albumin (BSA); Sigma) and incubated in 150 mM glycine (Merck; 10 min at room temperature (RT)), then with sodium borohydride (Sigma; 10 min), and finally in phosphate-buffered saline (PBS) containing 0.1 % Triton X-100 (Sigma). Preparations were incubated with primary antibody (see Additional file 1: Table S1) for 1 h at RT, washed, and incubated with the appropriate secondary antibody (1 h). Slides were incubated with Sytox Green and mounted in ProLong antifade reagent (both from Invitrogen). Images were captured with a Leica SP5, Zeiss LSM 700, or LSM 780 coupled to a two-photon Spectra-Physics Mai.Tai laser scanning confocal microscope, and were assembled with ImageJ (NIH). Processing, which included pseudo-color assignment and brightness changes, was applied uniformly across the entire image and used exclusively to equalize the appearance of multiple panels in a single figure.

### Cell isolation and flow cytometry

*Bmi1*-YFP hearts were perfused with PBS to remove blood cells and processed by digestion with 0.1 % collagenase IV (Sigma) and 10 μg/ml DNAse (Roche) (40 min, 37 °C). The resulting single cell suspension was passed through a 40-μm filter to remove debris. YFP^+^ cells were separated from the total heart mass on a BD FacsAria II cell sorter fitted with a 488 nm laser to excite YFP (collected in the 525/50 channel). To discriminate YFP^+^ from autoflorescent cells, a 488 nm laser was used to excite cells, followed by collection in the 585 channel (phycoerythrin). These peak emission wavelengths are far enough apart so that each signal can be detected by a separate detector.

For the *Sca-1* population, the CD45^+^ fraction was removed by discarding CD45^+^ cells using 405-conjugated rat anti-CD45 (1:100) and selecting for SCA-1 with APC-rat anti-SCA-1/Ly6a (1:100; both from BD Pharmingen). Data were analyzed using Facs DIVA Software.

### Isolation of adult mouse cardiomyocytes

Adult mouse CM were isolated from failing hearts of TM-induced adult *Bmi1*-YFP mice. The heart was rapidly removed and retrograde perfused under constant pressure (60 mmHg; 37 °C, 8 min) in Ca^2+^-free buffer (113 M NaCl, 4.7 mM KCl, 1.2 mM MgSO_4_, 5.5 mM glucose, 0.6 mM KH_2_PO_4_, 0.6 mM Na_2_HPO_4_, 12 mM NaHCO_3_, 10 mM KHCO_3_, 10 mM Hepes, 10 mM 2,3-butanedione monoxime, 30 mM taurine). Digestion was initiated by adding Liberase Blendzyme recombinant enzyme mix (0.2 mg/ml; Roche), trypsin (0.14 mg/ml; Invitrogen), and CaCl_2_ (12.5 μM) to the perfusion solution. When the heart became swollen and soft (10 min digestion) it was removed and gently teased into small pieces with fine forceps in the same enzyme solution. Heart tissue was further dissociated using a graded series of plastic transfer pipettes (2-, 1.5-, and 1-mm diameters openings) until all large pieces of heart tissue were dispersed in a cell suspension. Digestion buffer was neutralized with stopping buffer (10 % fetal bovine serum (FBS), 12.5 μM CaCl_2_). CM were pelleted by gravity (20 min), the supernatant aspirated, and CM resuspended in perfusion solution with 5 % FBS and 12.5 μM CaCl_2_. The calcium content was increased by gradual CaCl_2_ addition (62 μM to 1 mM). CM were pelleted by gravity (15–20 min) and, in culture dishes pre-coated with 0.5 g/ml mouse laminin (BD Biosciences) in PBS (1–2 h, RT), CM were plated in plating medium (Medium 199 Hank’s (Invitrogen), 0.25 % BSA (Sigma), 22 mM NaHCO_3_, 0.05 % FBS (Sigma), 0.001 % ITS Supplement (Gibco), 10 mM 2,3-butanedione monoxime, 25 μM blebbistatin (Sigma)) (2 h); CM were fixed with 2 % PFA and used for immunocytochemistry. In Fig. 4d and e, CM were plated, fixed and stained for SαA, GFP, and DAPI. The number of YFP^+^ CM and nuclei were assessed on duplicated microscope cover glasses (12 mm. diameter). At least 200 CM per cover glass were counted.

### In vivo proliferation analysis

EdU (5-ethynyl-2'-deoxyuridine; Sigma) was dissolved in 0.9 % NaCl solution and stored at 10 mg/ml. For proliferation experiments, mice received EdU (10 μg/g, i.p., once daily, 14 consecutive days). For the homeostatic group the pulse started on day 6 post-TM induction, and for the AMI group the pulse was given the day after AMI (day 6 post-TM induction).

After heart digestion (see above) YFP^+^ cells were sorted. For EdU staining, the Click-iT Plus EdU Alexa Fluor 647 Flow Cytometry reaction (Life Technologies) was used according to the manufacturer's instructions. Cell cycle was analyzed by flow cytometry with propidium iodide.

### RNA isolation and RNAseq analysis

RNA was extracted from hearts of 8-week-old TM-induced *Bmi1*-YFP mice. In the homeostatic study at 5 days post-TM induction RNA was extracted from 4 × 10^5^ sorted *Bmi1*-CPC (YFP^+^) and *Sca-1*-YFP^–^ cells from four replicates each (6 mice/replicate). Under injury conditions AMI was performed 5 days post-TM induction, and 5 days post-AMI RNA was extracted from 4 × 10^5^ sorted *Bmi1*-CPC (YFP^+^) cells from four replicates (2–3 mice/replicate).

Total RNA was isolated with the Arcturus PicoPure kit (Applied Biosystems). cDNA was amplified from total RNA (10–25 ng) using the Ovation RNA-seq System v2 (NuGEN Technologies). Amplified cDNA (1 μg) was sonicated to an average size of 200–300 bp and used with the TruSeq DNA Sample Preparation v2 Kit (Illumina) to generate index-tagged sequencing libraries. Library quality and size distribution were determined with the Agilent Bioanalyzer DNA-1000 Kit (Agilent Technologies) and quantified with the Nanoquant spectrophotometer (Tecan). Libraries were applied to an Illumina flow cell for cluster generation (True Seq SR Cluster Kit V2 cBot). Single reads of 75 bases were generated on the Genome Analyzer IIx using the TruSeq SBS Kit v5 (Illumina) following standard sequencing protocol. Ingenuity Pathway Analysis (IPA) software was used to integrate RNAseq analysis data to define plausible signaling cascades by autogenerating regulatory networks. Gene Ontology (GO) heat maps included significant term analysis (adjusted *P* value <0.05) of genes differentially expressed in *Bmi1*-CPC versus *Sca-1*-YFP^–^ cardiac compartments. GO analysis of significantly over- and under-represented terms was conducted with FatiGO (www.babelomics.org). Heat maps in Fig. 3d were obtained with Genesis software.

### Histology (hematoxylin/eosin staining)

Neonatal hearts from *Bmi1*-YFP mice at 1, 7, and 21 days post-resection (dpr) were rinsed with PBS, embedded directly in OCT (Tissue Tek), and stored at –80 °C. Cryosections (8 μm) were hematoxylin/eosin-stained following standard procedures.

### Cardiac injury models in neonatal and adult mice

The left ventricular apex was resected in neonatal hearts from P1 *Bmi1*-YFP mice. Neonates were anesthetized by cooling on an ice bed (4 min). Lateral thoracotomy at the fourth intercostal space was performed by blunt dissection of the intercostal muscles following skin incision. To separate the ribs we used 1-mm wide retractors (FST) to expose the heart. Iridectomy scissors were used to resect the P1 heart apex to expose the left ventricular chamber. Thoracic wall incisions were sutured with 7-0 non-absorbable silk suture, and the skin wound closed using skin adhesive (Vetbond, 3M). Sham-operated mice underwent the same procedure without apical resection. Neonates were then placed under a heat lamp and warmed for several minutes until recovery.

For AMI in adult hearts, mice were anesthetized with 4 % sevoflurane, intubated, and ventilated with a 50 % air:oxygen mixture using a positive-pressure respirator (Minivent 845, Harvard; 160 strokes/min, 250 μl tidal volume). A left thoracotomy was performed via the fourth intercostal space and the lungs retracted to expose the heart. After opening the pericardium, the left anterior descending coronary artery was ligated with 7-0 silk suture approximately 2 mm below the edge of the left atrial appendage. Ligation was considered successful when the anterior wall of the left ventricle turned pale. The lungs were inflated by increasing positive end-expiratory pressure and the thoracotomy site closed in layers with 6-0 suture. Animals were maintained on a 37 °C heating pad until recovery and for 2 h after surgery. Another group of mice underwent sham ligation, with a similar surgical procedure without tightening the suture around the coronary artery.

### Statistical analysis

Statistical analysis was performed with GraphPad Prism 5.0. Significance between groups was evaluated in all experiments as detailed for each figure. A value of *P* < 0.05 was considered significant. All replicates considered are biological replicates. *P* values were calculated by unpaired Student’s *t* test with Welch’s correction. Data are shown as mean ± SEM.

## Results

### Transcriptome study of *Bmi1*-CPC isolated from unchallenged hearts shows enrichment in stemness-related genes

We previously reported that non-myocyte *Bmi1*-CPC contribute to homeostatic cardiomyocyte turnover in the adult murine heart [15]. Following TM induction of healthy *Bmi1*^CreER/+^;*Rosa26*^YFP/+^ mice (*Bmi1*-YFP), flow cytometry of non-cardiomyocyte heart cells identified a YFP-expressing population (2.7 ± 0.2 % cells/heart) 5 days post-TM induction that represents a small subset (5.4 ± 0.4 %) of the SCA-1^+^ cardiac population [15]. Additionally, lineage tracing of *Bmi1*^+^-derived progeny up to 1 year after TM induction showed YFP^+^ CM (6.7 ± 1.3 %) in vivo.

**Fig. 1:**
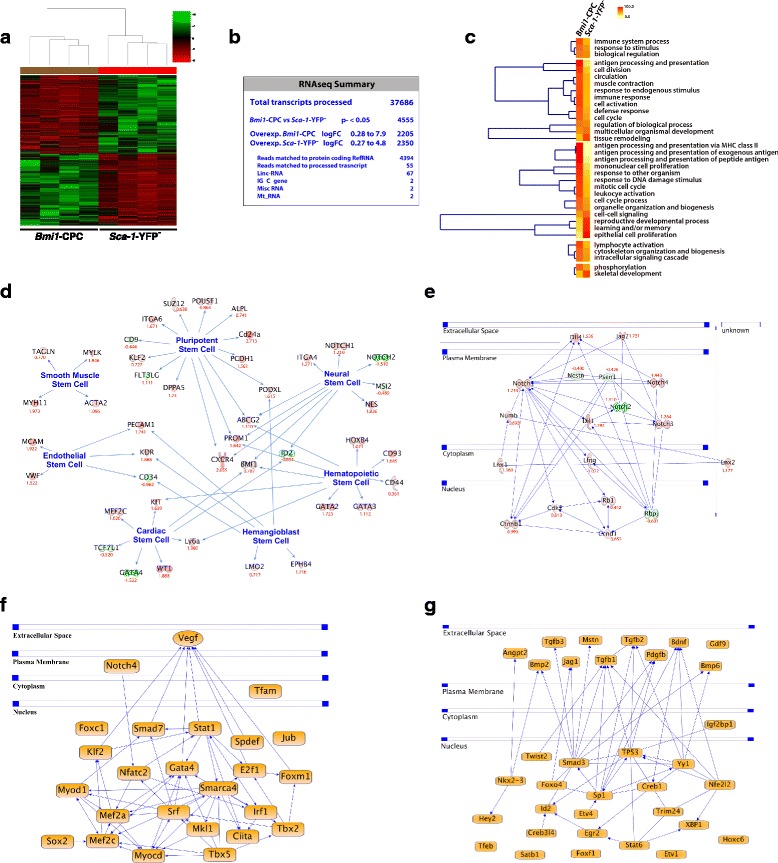
*Bmi1*-CPC have a stemness profile and upstream regulators related to cardiac development and pluripotency. **a** Heat map of matched samples (*n* = 4 replicates; 6 mice/replicate) in high-throughput RNAseq analysis of *Bmi1*-CPC and *Sca-1*-YFP^–^ cells. **b** RNAseq gene summary. **c** Gene Ontology (GO) heat map including significant terms analysis (adjusted *P* < 0.05) of genes differentially expressed in *Bmi1*-CPC versus *Sca-1*-YFP^–^ compartments. GO analysis of significantly over- and under-represented terms with FatiGO. **d** Pluripotency pathway analysis. Comparative expression of stemness-related genes. *Red* indicates significant overexpression in *Bmi1*-CPC and *green* significant overexpression in *Sca-1*-YFP^–^ populations. Numbers in *red* represent logarithmic fold change (log FC). **e** IPA analysis of the Notch pathway in *Bmi1*-CPC versus *Sca-1*-YFP^–^ populations. *Red* indicates significant overexpression in *Bmi1*-CPC and *green* significant overexpression in *Sca-1*-YFP^–^ populations. Numbers in *red* represent log FC. **f** IPA prediction for main upstream regulators in the *Bmi1*-CPC population. **g** IPA prediction for main upstream regulators in the *Sca-1*-YFP^–^ population

To define the *Bmi1*-CPC genetic profile more precisely, we conducted a comparative RNAseq analysis of *Bmi1*-CPC (YFP^+^) and *Sca-1*^+^, YFP-negative reference (*Sca-1*-YFP^–^) cell populations sorted from hearts of 6- to 8-week-old *Bmi1*-YFP mice 5 days post-TM induction. Heat map analysis differentiated the two populations and defined precise profiles for each (Fig. 1a). Of a total of 37,686 transcripts processed, 4555 (12 %) were differentially expressed between the *Bmi1*-CPC and *Sca-1*-YFP^–^ populations (Fig. 1b). GO analysis showed high representation of genes related to immunity, circulation, and muscle contraction in the *Bmi1*-CPC (Fig. 1c). The *Bmi1*-CPC expression profile overlapped with other stem cell systems, including expression of critical embryonic and adult multipotent markers (*Pou5f1, Suz12, Dppa5*, *Prom1*) (Fig. 1d) which was not shared by the *Sca-1*-YFP^–^ cells. The Notch pathway, which is implicated in cardiac development, was upregulated in the *Bmi1*-CPC compartment (Fig. 1e). Additionally, IPA of upstream regulators in the *Bmi1*-CPC population predicted enrichment for functions related to stemness (*Sox2, Klf2*) and cardiac development (*Srf, Myocd*, *Tbx5*) (Fig. 1f). Conversely, the main upstream regulators predicted by IPA in the *Sca-1*-YFP^–^ population were growth factors (Fig. 1g). These findings suggest that *Bmi1*-CPC have the potential to contribute to cardiac fate while remaining competent for self-maintenance.

### *Bmi1*-CPC are activated in response to acute myocardial infarction

**Fig. 2:**
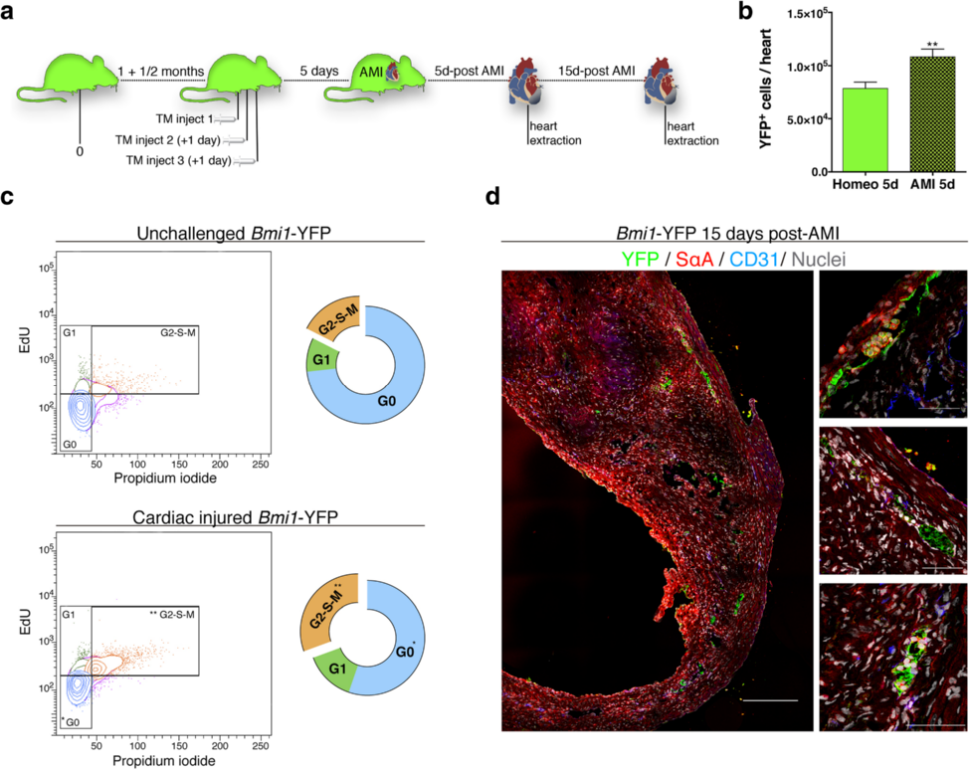
Activation of *Bmi1*-CPC after acute myocardial infarction. **a** Timeline for analysis of *Bmi1*-CPC cells and progeny. **b** Number of YFP^+^ cells obtained by FACS sorting 5 days post-acute myocardial infarction (*AMI*; *n* = 9) compared to the uninjured heart (homeostatic) condition (*n* = 15). **P < 0.01. Data shown as mean ± SEM. **c** Cell cycle study of freshly isolated YFP^+^ cells after EdU administration to *Bmi1*-YFP mice in physiological (homeostasis; *top*) and post-AMI conditions (*bottom*) (*n* = 4). *P < 0.05, **P < 0.01. **d** Partial transverse section of an injured *Bmi1*-YFP left ventricle 15 days post-AMI (*left*) and detailed YFP^+^ clusters located in the infarcted area (*right*). *Scale bars* = 200 μm (*left*), 50 μm (*right* panels)

To evaluate the *Bmi1*-CPC-mediated response after heart injury, we conducted lineage-tracing analysis in adult *Bmi1*-YFP mice (8–12 weeks old) that underwent AMI at 5 days post-TM induction. *Bmi1*-CPC-derived progeny (YFP^+^ cells) were traced after Cre-mediated recombination of the responder cassette in *Bmi1*-YFP mice (Fig. 2a). Five days after AMI, the number of YFP^+^ cells isolated from injured *Bmi1*-YFP mouse hearts was significantly larger (≈1.5-fold) than in healthy (homeostatic) littermates (Fig. 2b). This finding was supported by in vivo pulse-chase EdU labeling of YFP^+^ cells, which compared physiological and AMI conditions. The YFP^+^ population had a higher proliferation rate (≈2-fold) after AMI (Fig. 2c). Immunohistochemistry of heart sections at 15 days post-AMI detected YFP^+^ clusters in niche-like structures near and within the infarcted zone (Fig. 2d).

### Cardiac injury induces an activated phenotype in *Bmi1*-CPC

**Fig. 3:**
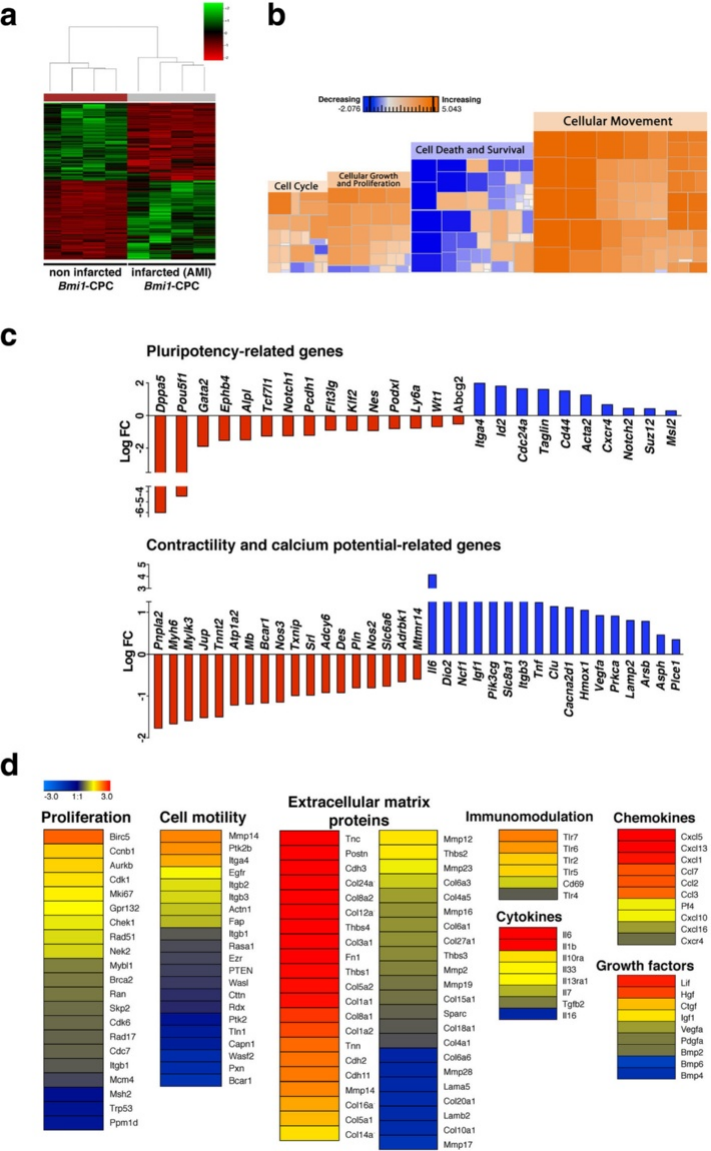
*Bmi1*-CPC upregulate pathways related to proliferation and cell motility and downregulate some pluripotent genes after cardiac injury. **a** Heat map of matched samples (*n* = 4 replicates, 2–3 mice/replicate) in the high-throughput RNAseq analysis of sorted *Bmi1*-CPC (YFP^+^) cells from adult TM-induced control mice (non-infarcted) and mice subjected to acute myocardial infarction (*AMI*) at 5 days post-treatment. **b** Heat map of molecular and cellular functions in *Bmi1*-CPC 5 days post-AMI, compared to counterparts from non-infarcted hearts, and classified by activation z-score. **c** Differential expression of genes implicated in pluripotency and muscle contractility and calcium management. Genes are classified as down- (*red*) and upregulated (*blue*) in sorted *Bmi1*-CPC 5 days post-AMI by comparison with *Bmi1*-CPC counterparts from non-infarcted hearts. **d** Heat map of proliferation and cell migration functions and genes related to the extracellular compartment, immunomodulation, cytokines, chemokines, and growth factors in sorted *Bmi1*-CPC 5 days post-AMI by comparison with *Bmi1*-CPC counterparts from non-infarcted hearts. Color scale intensity is related to gene log fold change (*FC*)

To evaluate the changes in mRNA transcript levels in adult *Bmi1*-CPC after severe cardiac injury, we induced AMI in adult *Bmi1*-YFP mice (6 to 8 weeks old) 5 days post-TM induction. YFP^+^ cells were isolated from hearts at 5 days post-AMI and the transcriptome analyzed by RNAseq. In comparison with *Bmi1*-CPC isolated from control, non-infarcted *Bmi1*-YFP mice, the analysis showed differential expression of more than 5500 genes; heat map analysis neatly differentiated the two conditions (Fig. 3a). IPA showed post-injury upregulation of genes related to cell growth and proliferation (1574 genes), cell death and survival (1533), cell movement (1001), and cell cycle (727) (Fig. 3b). This activation was accompanied by clear dysregulation of several multipotency markers such as *Dppa5* and *Pou5f1*, as well as genes related to muscle contractility and calcium management (*Pnpla2*, *Myh6*) (Fig. 3c). Key transcripts involved in pathways such as cell proliferation, movement, and cell cycle were significantly upregulated in *Bmi1*-derived cells 5 days post-AMI (Fig. 3d). *Bmi1*-CPC after AMI also showed marked upregulation of extracellular matrix and several growth factors, and pro- and anti-inflammatory cytokines were also notably upregulated (Fig. 3d).

These results suggest a proliferative activation of *Bmi1*-derived cells shortly after AMI, and the upregulation of crucial cell functions that are required to respond after tissue injury and instigate repair.

### *Bmi1*-CPC contribute to cardiac repair with de novo cardiomyocytes after myocardial infarction in adult mice but not after neonatal apical heart resection

**Fig. 4:**
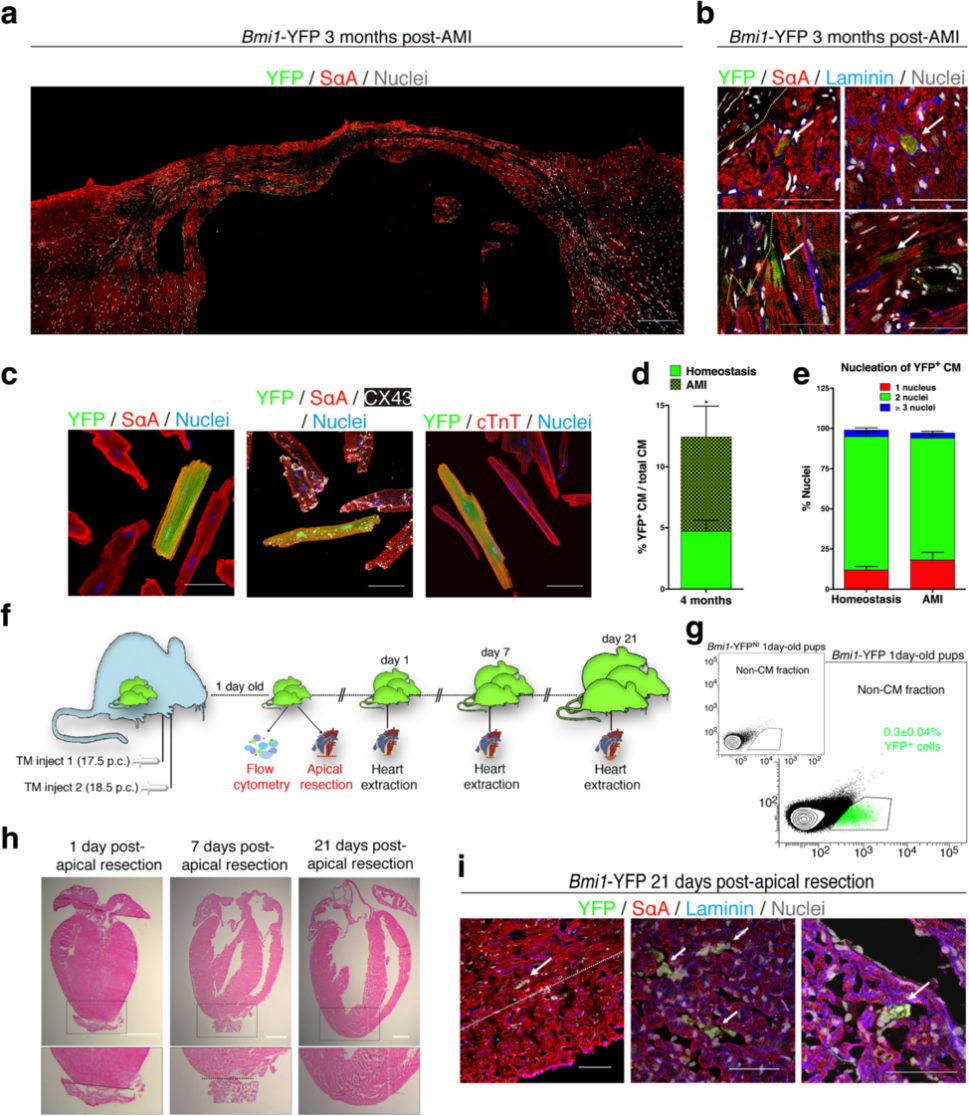
*Bmi1*-CPC response to acute injury in adult and neonatal mouse hearts. **a** Detailed transverse section of *Bmi1*-YFP left ventricle 3 months post-acute myocardial infarction (*AMI*). *Scale bar* = 200 μm. **b** YFP^+^ cardiomyocytes (*CM*) 3 months post-AMI, located near the injured area (*left*) and at sites distant from the infarcted zone (*right*); (*n* = 3). *Dotted line* delineates the border of the injured area. *Arrows* indicate YFP^+^ CM. *Scale bars* = 50 μm. **c** Adult CM freshly isolated 4 months post-AMI. YFP^+^ CM express cardiac proteins related to contractility and gap junctions, including sarcomeric α-actinin (*SαA*), (cardiac troponin T (*cTnT*), and connexin 43 (*CX43*). *Scale bars* = 50 μm. **d** Number of YFP^+^ CM in adult mice (4 months post-AMI, *n* = 4) compared with the number in non-infarcted hearts (homeostasis; 4 months post-AMI, *n* = 4). *P < 0.05. Data shown as mean ± SEM. CM were plated, fixed, and stained for SαA, GFP, and DAPI. The number of YFP^+^ CM was assessed in duplicated microscope cover glasses (12 mn diameter). At least 200 CM per cover glass were counted. **e** Nucleation of YFP^+^ CM in homeostasis and after AMI (n = 3-4 mice). Data shown as mean ± SEM. CM were isolated 4 months post-AMI, plated, fixed and stained for SαA, GFP and DAPI. Nucleation of YFP^+^ CM was assessed in duplicated microscope cover glass (12 mm diameter). At least 200 CM per cover glass were counted. **f** Schematic representation of apical resection experiments on tamoxifen (*TM*)-induced neonatal hearts. **g** YFP^+^ fraction in postnatal day 1 (P1) *Bmi1*-YFP hearts accounts for 0.3 ± 0.04 % of total mononuclear heart cells (*n* = 8). YFP^+^ cells were not detected in non-induced controls (*Bmi1*-YFP^NI^, inset). Data shown as mean ± SEM. **h** Hematoxylin/eosin staining of mouse heart at 1, 7, and 21 days post-apical resection (dpr) in neonatal (P1) mouse pups. *Dashed line* delineates the resection plane. Enlarged view of resected areas is shown, delimited by *dashed squares. Scale bars* = 1000 μm. **i** Immunodetection of YFP^+^ clusters (*arrows*) near the injured area (*left* and *center*) and atrium (*right*) 21 days after neonatal heart apical resection (*n* = 3). *Dashed line* delimits the regenerated apex 21 dpr. *Scale bars* = 50 μm (*left*, *right*), and 20 μm (*center*)

To evaluate the response of *Bmi1*^+^-derived cells at late stages of cardiac injury, we traced the progeny of *Bmi1*-CPC at 3 months post-AMI. Although YFP^+^ clusters were virtually undetectable in the infarcted zone (Fig. 4a), immunohistochemical analysis found YFP^+^ CM (as determined by the marker sarcomeric α-actinin (SαA)) located predominantly at the infarct border and in remote zones (Fig. 4b), but not in the area of injury. Immunostaining for a panel of contractility-related proteins including cardiac troponin T, SαA, and the gap junction protein, connexin 43, in isolated CM 4 months post-AMI showed that YFP^+^ CM were indistinguishable from YFP^–^ CM (Fig. 4c). We also assessed the number of YFP^+^ CM isolated from *Bmi1*-YFP mice at 4 months post-AMI; these cells made up 13.8 ± 5 % of total CM compared to 4.7 ± 0.9 % in age-paired non-infarcted hearts (Fig. 4d). YFP^+^ CM exhibit the same nucleation pattern as the YFP^–^ CM (Fig. 4e), which are mainly binucleated (80 %). These data reinforce the hypothesis that *Bmi1*-CPC are a reservoir of adult cardiac progenitor cells able to respond to acute injury with de novo generation of CM.

Additionally, the relevant regenerative capacity that the mouse neonatal heart exhibits during the first week of life has been recently reported [16, 17]. Therefore, we next explored the potential role of *Bmi1*-CPC in these early cardiac stages (Fig. 4f). We treated pregnant mice (*Bmi1*^*CreER*/CreER^ or *Rosa26*^YFP/YFP^ females) with TM (E17.5, E18.5); flow cytometry confirmed YFP expression in neonatal heart cells (double heterozygotes) at postnatal day 1 (P1) (Fig. 4g). *Bmi1*-YFP pups that underwent apex resection at P1 were sacrificed at P21, when the apex was fully restored (Fig. 4h). YFP^+^ cells were detected in the atrium and ventricle, but very rarely or not at all in the regenerated area (Fig. 4i); this indicates that *Bmi1*-derived cells make no major contribution to immediate postnatal regeneration.

## Discussion

Our study provides evidence that *Bmi1*-derived cells contribute to de novo generation of CM after myocardial infarction, which is greater than for heart homeostasis with ageing. These findings consolidate *Bmi1*-CPC cells as a sound source of progenitors for cardiac repair.

The cardiac stem field was initiated by the isolation and characterization of *c-KIT*^+^ CPC [9], which recently were proposed necessary and sufficient for heart repair after injury [18]. *c-kit* expression in vitro and in vivo has yielded disparate results, however, which probably reflects the extremely variable expression of this marker in distinct contexts and conditions [19, 20]. Three recent independent lineage-tracing studies found that *c-kit*^+^ cardiac progenitor cells produce <0.008 % of the new CM in adult mouse heart, and proposed that *c-kit*^+^ cells are not relevant either for homeostasis or after myocardial infarction [21–23]. Our own research on *Bmi1*-CPC showed that these progenitors are mainly negative for c-KIT expression (0.1–1 %) [15], suggesting that the majority of de novo CM derive from *Bmi1*^+^*c-kit*^–^ progeny.

Putative CPC have also been isolated based on their expression of the established hematopoietic marker SCA-1 [24], although SCA-1 appears to label a heterogeneous population with predominantly endothelial potential [25, 26]. Genetic elimination of *Sca-1* affects resident CPC, which then fail to respond to pathological damage in vivo; this coincided with impaired in vitro growth and survival of these cardiac progenitor cells [27]. SCA-1 CPC contributes to CM generation in a model of pressure overload cardiac injury (transverse aortic constriction), but not after AMI [24]. Our *Bmi1*-CPC showed a strong response after AMI and the percentage of de novo CM generated is greater than that observed during normal cardiac homeostasis. Although Uchida et al*.* [24] found no major contribution by the *Sca-1*^*+*^ population in new CM formation after acute injury, the distinct methods and transgenic models used in these studies could explain the differences. The authors nonetheless suggested that only a small fraction of the *Sca-1*^*+*^ population contributes to the CM lineage [24]. *Bmi1*-CPC make up a fraction of the *Sca-1*^*+*^ population [15], and our results here suggest that *Bmi1*^*+*^ cells are the *Sca-1*^+^ subset involved in this new CM generation. The information gleaned from the deep-sequencing analysis of *Bmi1*-CPC from healthy hearts reinforces the idea that this population expresses higher levels of pluripotency markers. We consider *Bmi1* to be a key transcription factor that controls stemness in the adult heart, thus defining a population of cardiac progenitors. This would be in agreement with the critical positive role of *Bmi1* in fibroblast reprogramming to embryonic stem cells [28, 29] and the very recent description as a key epigenetic barrier to direct cardiac reprogramming [30].

The limited capacity of the adult mammalian heart to recover after myocardial injury is well established. A genetic fate-mapping strategy gave indirect evidence that up to 19 % of CM are replaced 3 months post-AMI, but the source of the new CM was not definitively determined [31]. Our lineage-tracing studies after cardiac infarction show that *Bmi1*-expressing cells contribute to significant and comparable numbers of de novo CM. This result provides direct evidence of a resident cardiac progenitor population that contributes to heart repair. RNAseq analysis of *Bmi1*-CPC isolated 5 days after AMI showed an increase in genes related to cell proliferation, movement, and cell cycle functions, all of which are necessary for cells to reach the infarcted area and to instigate repair. *Bmi1*-derived cells also showed marked upregulation of extracellular matrix proteins, growth factors, and pro- and anti-inflammatory cytokines. The data from the RNAseq study indicate that, following cardiac injury, *Bmi1*-CPC sense the unfavorable environment originated by the insult and respond by upregulating pathways related to key cell functions required to promote repair. Although we did not detect a defined pattern of cardiomyocyte specification markers in the *Bmi1*-derived cells 5 days after injury, surviving *Bmi1*-CPC at this early stage might need to proliferate and migrate before they can respond appropriately to injury-induced signals. Lineage tracing of *Bmi1*^*+*^ cells at 4 months post-AMI showed generation of 13.8 ± 5 % new YFP^+^ CM, which coincides with some previous reports [31] and pinpointed the *Bmi1*-CPC ability to contribute to myocardial repair after injury. Immunostaining of a panel of contractility-related proteins showed that these YFP^+^ CM were indistinguishable from YFP^–^ CM. Although we did not detect YFP^+^ CM within the injured area, these new CM might have an important role in supporting basal heart beat and cardiac function after AMI. In any case, the role of *Bmi1*^+^-CPC in cardiac repair after AMI is compatible with contributions from other reported sources [24, 32]. Further characterization of the mechanisms that lead to endogenous progenitor cell activation and of the mechanisms that permit repopulation of the infarcted region could be a new opportunity for therapeutic applications.

Although the adult mammalian heart is one of the least regenerative organs in the body, different studies have described heart regeneration in lower vertebrates and neonatal mammals following apical resection [33, 34]. Lineage tracing experiments in adult zebrafish following cardiac injury suggest that the vast majority of the regenerated CM are derived from pre-existing proliferative CM rather than from a population of cardiac stem/progenitor cells [34, 35]. Additionally, the neonatal mouse heart has shown a similar ability to restore the lost myocardial tissue within the first week of postnatal life [17, 36, 37], primarily, by proliferation of pre-existing cardiomyocytes and not by putative population(s) of endogenous CPC [38, 39]. However, the ability to efficiently regenerate the heart muscle upon injury is essentially lost by postnatal day 7, coinciding with the developmental window when mouse CM mostly binucleate and withdraw from the cell cycle [40]. This compromised ability to fully repair the damaged/resected myocardium is mainly related to the lack of basal proliferation in adult CM. However, there are several reports pinpointing the relevant role of endogenous CPC leading to the reparative response in the adult mouse following cardiac injury [24, 31, 32, 41]. Although adult CPC do not fully restore a severe acutely damaged tissue, their contribution to de novo CM may contribute to maintain organ functionality. Altogether these reports, and our own data (apical resection of TM-induced day 1 *Bmi1*-YFP pups indicate a minor contribution of *Bmi1*-derived cells to de novo CM), may suggest a switch in CPC reparative role through the lifespan of the organism.

Therefore, further studies will determine and evaluate the mechanisms by which *Bmi1*^+^ cells remain less active in neonatal heart regeneration but play a more direct role in adult heart repair. Moreover, it will also be necessary to understand the hypothetic crosstalk existing between CM and *Bmi1*^+^ through the lifespan of the animal to clarify the different reparative responses that *Bmi1*^+^ exhibit following cardiac injury in the neonatal and adult heart.

## Conclusions

Our results show that *Bmi1* expression defines a multipotent cardiac cell population with capacity for myocardial repair following cardiac injury in adult mice. Future research to better characterize the biology of *Bmi1*-CPC will help to identify critical factors that allow their potential to be harnessed for effective cardiac cell therapy.

## Abbreviations

AMI, acute myocardial infarction; *Bmi1*, B cell-specific Moloney murine leukemia virus integration site 1; BSA, bovine serum albumin; EdU, 5-ethynyl-2'-deoxyuridine; c-KIT, Kit oncogene; CM, cardiomyocytes; Cre-ER, variant of the site-specific (loxP) recombinase Cre that binds to the estrogen receptor module (ER); CPC, cardiac progenitor cells; FACS, fluorescence-activated sorting; FBS, fetal bovine serum; GFP, green fluorescent protein; GO, gene ontology; i.p., intraperitoneal; IPA, ingenuity Pathway Analysis; PBS, phosphate-buffered saline; PFA, paraformaldehyde; RNAseq, RNA sequencing; Rosa26, mouse locus used for constitutive, ubiquitous gene expression; RT, room temperature; SαA, sarcomeric α-actinin; SCA-1, stem cell antigen-1; TM, tamoxifen; YFP, yellow fluorescent protein

## Additional file

Additional file 1: Table S1. Antibodies used in immunodetection. Antibodies used in this study. (JPG 199 kb)
