# Supplementary figures and images for: Bmi1+ cardiac progenitor cells contribute to myocardial repair following acute injury
Source: Stem Cell Res Ther. 2016 Jul 30;7:100. doi: 10.1186/s13287-016-0355-7 (PMC4967328; doi:10.1186/s13287-016-0355-7)

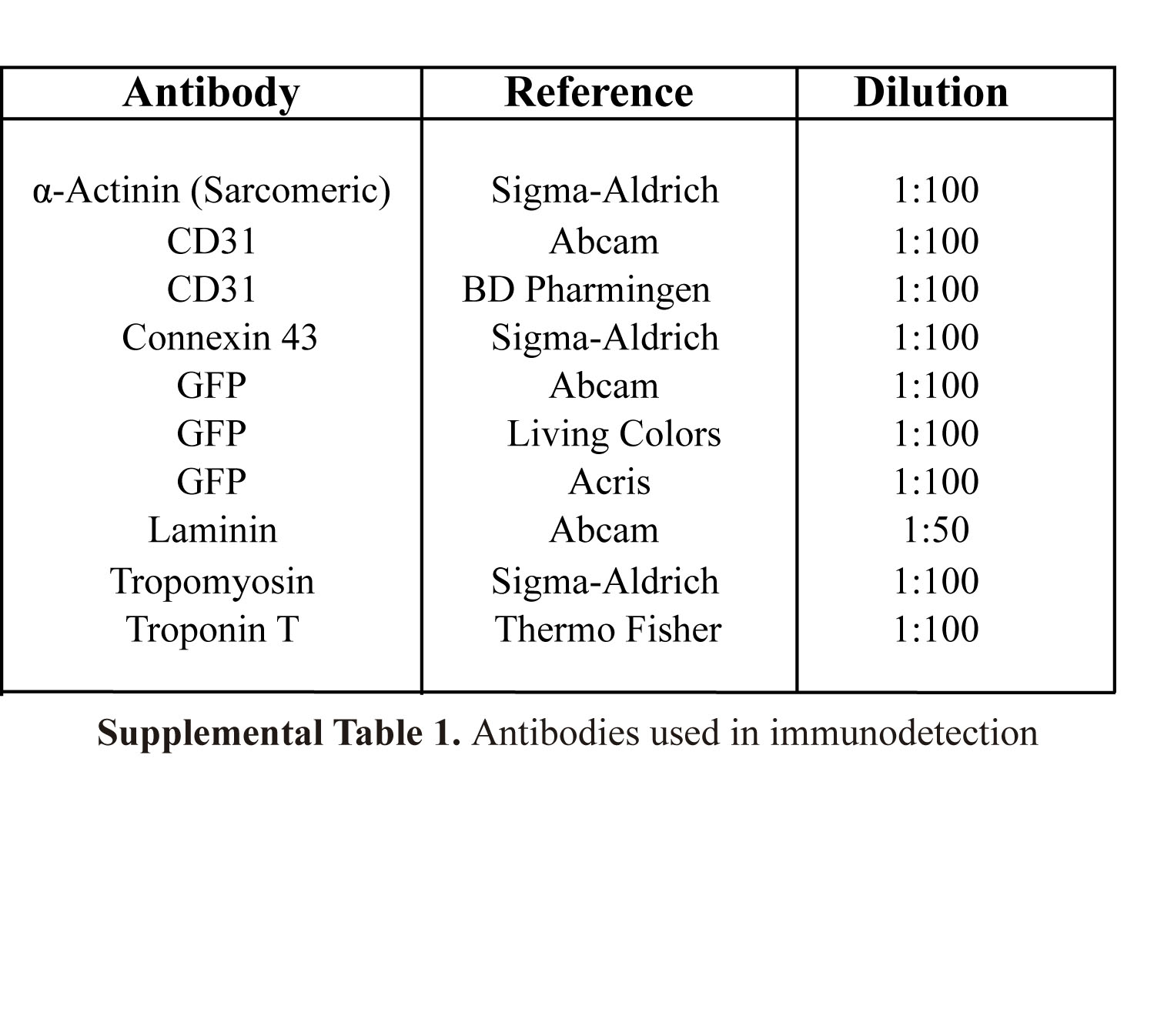

Supplement: Additional file 1: Table S1. — Antibodies used in immunodetection. Antibodies used in this study. (JPG 199 kb) [file 13287_2016_355_MOESM1_ESM.jpg]
